# Risk stratification in cardiogenic shock: a focus on the available evidence

**DOI:** 10.1007/s10741-021-10140-7

**Published:** 2021-07-14

**Authors:** C. Sciaccaluga, G. E. Mandoli, N. Ghionzoli, F. Anselmi, C. Sorini Dini, F. Righini, F. Cesareo, F. D’Ascenzi, M. Focardi, S. Valente, M. Cameli

**Affiliations:** Department of Medical Biotechnologies, Section of Cardiology, University of Siena, Siena, Italy

**Keywords:** Cardiogenic shock, Risk stratification, Echocardiography, Biomarkers, Prognostic score

## Abstract

Cardiogenic shock is a clinical syndrome which is defined as the presence of primary cardiac disorder that results in hypotension together with signs of organ hypoperfusion in the state of normovolaemia or hypervolaemia. It represents a complex life-threatening condition, characterized by a high mortality rate, that requires urgent diagnostic assessment as well as treatment; therefore, it is of paramount important to advocate for a thorough risk stratification. In fact, the early identification of patients that could benefit the most from more aggressive and invasive approaches could facilitate a more efficient resource allocation. This review attempts to critically analyse the current evidence on prognosis in cardiogenic shock, focusing in particular on clinical, laboratoristic and echocardiographic prognostic parameters. Furthermore, it focuses also on the available prognostic scores, highlighting the strengths and the possible pitfalls. Finally, it provides insights into future direction that could be followed in order to ameliorate risk stratification in this delicate subset of patients.

## Introduction

Cardiogenic shock (CS) is a clinical syndrome defined by the presence of a primary cardiac disorder resulting in hypotension (systolic blood pressure < 90 mmHg, or vasopressors required to achieve a systolic blood pressure ≥ 90 mmHg) and signs of organ hypoperfusion (such as altered mental status, oliguria, cold and clammy skin and extremities, increased arterial lactate above 2 mmol/L) in the state of normovolaemia or hypervolaemia [1, 2]. Although not mandatory, objective haemodynamic parameters for CS, such as cardiac index and pulmonary capillary wedge pressure, might help confirm the diagnosis and outline the CS phenotype. Table 1 shows the different CS definitions according to the main trials and the latest guidelines.

**Table 1:** Definitions of cardiogenic shock

ESC Guidelines [19]	SHOCK trial [14]	IABP-SHOCK II trial [33]	CULPRIT-SHOCK Trial [78]	IMPRESS Trial [26]	SCAI [6]
Clinical criteria					
SBP < 90 mmHg despite adequate volume AND Clinical hypoperfusion: • Oliguria • Cold extremities • Mental confusion • Narrow pulse pressure OR Laboratory hypoperfusion: • Elevated serum lactate • Elevated serum creatinine • Metabolic acidosis	SBP < 90 mmHg for ≥ 30 min OR SBP ≥ 90 mmHg with support AND Evidence of hypoperfusion: urine output < 30 ml/h cold extremities	SBP < 90 mmHg for ≥ 30 min OR SBP > 90 mmHg with catecholamines AND clinical pulmonary congestion AND • Impaired end-organ perfusion (≥ 1): • Altered mental status • Cold/clammy skin and extremities • Urine output < 30 ml/h • Serum lactate levels > 2 mmol/L	SBP ≤ 90 mmHg for > 30 min OR Catecholamines required to maintain SBP > 90 mmHg AND Pulmonary congestion AND • Impaired end-organ perfusion (≥ 1): • Altered mental status • Cold/clammy skin and extremities • Urine output < 30 ml/h • Serum lactate levels > 2 mmol/L	SBP ≤ 90 mmHg for > 30 min OR SBP > 90 mmHg with vasopressors/inotropes	SBP < 90 mmHg or MAP < 60 mmHg OR SBP drop > 30 mmHg OR Inotropy/support to maintain SBP ≥ 90 mmHg or MAP ≥ 60 mmHg • Volume overload • Extensive rales • Killip class 3 or 4 • BiPap or mechanical ventilation • Cold, clammy acute alteration in mental status • Urine output < 30 mL/h • Lactate ≥ 2 • Creatinine doubling or > 50% drop in GFR • Increased LFTs • Elevated BNP
Haemodynamic criteria					
	• CI < 2.2 L/min/m2 AND • PCWP > 15 mmHg				• CI < 2.2 L/min/m2 • PCWP > 15 mmHg • RAP/PCWP ≥ 0.8 • PAPI < 1.85 • Cardiac power output ≤ 0.6

CS is a complex life-threatening condition requiring urgent assessment and treatment. In fact, the short-term mortality of CS remains particularly high, currently attested to be around 40% [3], even though the use of more aggressive and invasive strategies has raised over the last decade.

### Classification of cardiogenic shock

**Fig. 1:**
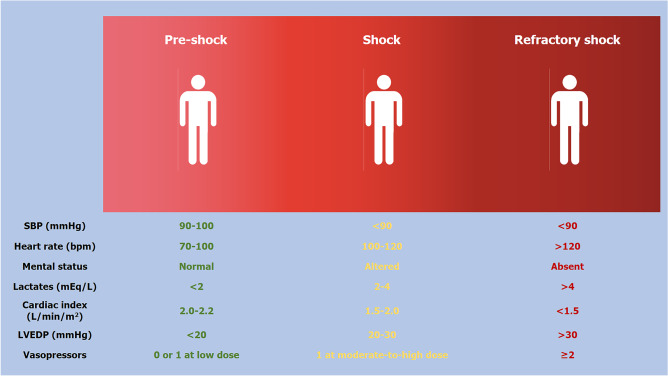
Cardiogenic shock phenotypes

**Fig. 2:**
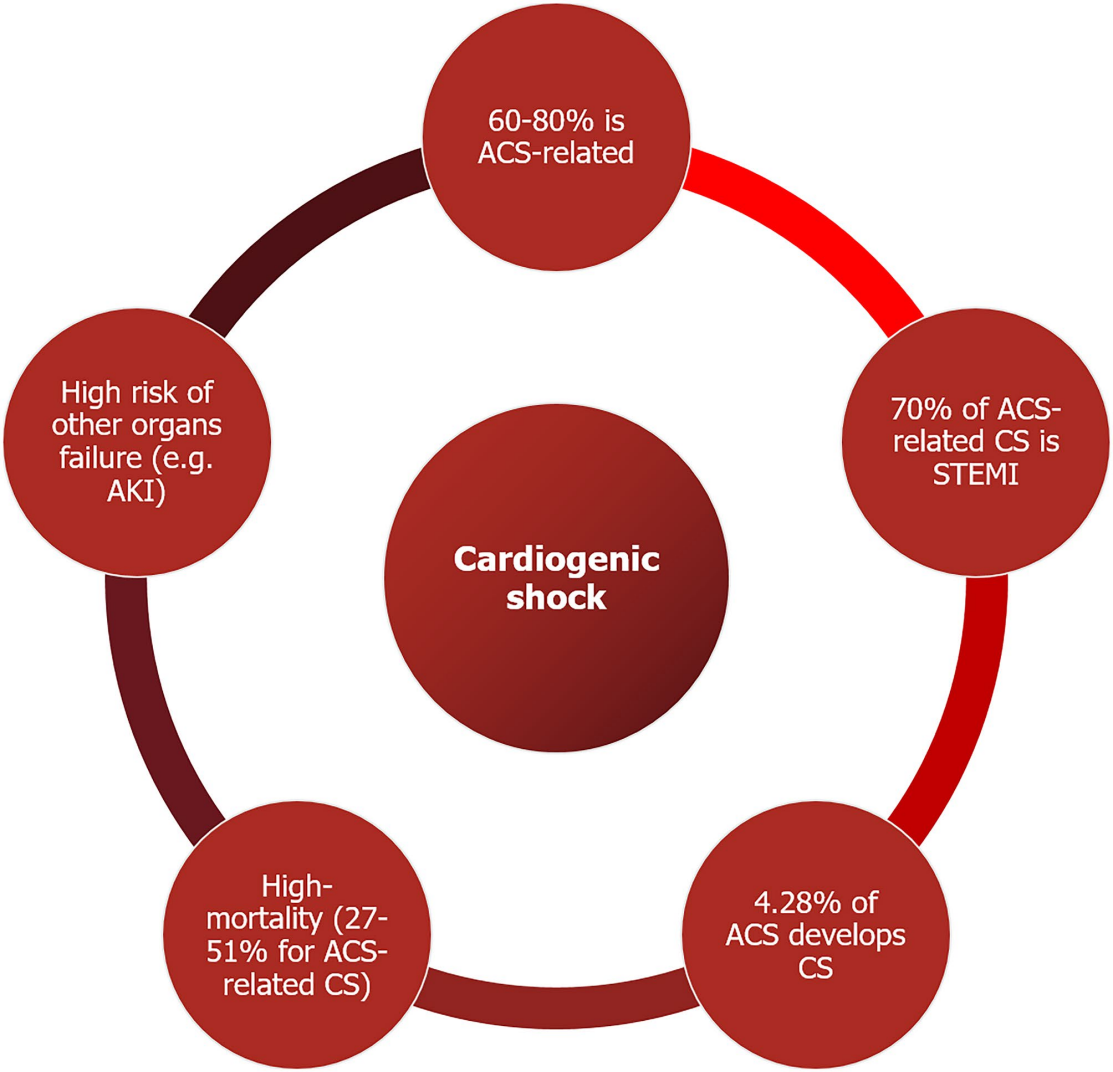
Cardiogenic shock epidemiology

According to its pathophysiological cascade, CS might be divided into three phases: ‘pre-shock’ phase, in which there is evidence of hypoperfusion even if systolic blood pressure is > 90 mmHg thanks to the increased peripheral vascular resistance; ‘shock’ phase where hypoperfusion and hypotension coexist; and ‘refractory shock’ phase in which the hypoperfusion is unresponsive to the adopted strategies (Fig. 1) [4, 5]. The identification of the patient’s stage is key to provide the best management. Another aspect to consider in the overview of CS is the underlying cause of the cardiac dysfunction. In fact, despite the fact that CS is a heterogeneous syndrome, its aetiology can be basically divided into two major groups: acute coronary syndrome (ACS)-related CS and non-ACS-related CS. In view of improving CS patients’ risk stratification, the Society for Cardiovascular Angiography and Interventions (SCAI) proposed a new classification of CS, described as Interagency Registry for Mechanically Assisted Circulatory Support (INTERMACS) profile 1, attempting to overcome the heterogeneity of patients [6]. This classification includes 5 stages labelled A–E (Fig. 2): stage A ‘at risk’ for developing CS, stage B ‘beginning’ CS (pre-shock) when the patient presents hypotension without hypoperfusion, stage C ‘classic’ CS indicating the coexistence of hypotension with hypoperfusion, stage D ‘deteriorating’ CS in which further escalation of therapy is required and stage E ‘Extremis’ CS that is circulatory collapse; a modifier ‘_A_’ is also considered, indicating that the patient had a cardiac arrest [6]. SCAI classification has been recently validated in retrospective studies [7, 8], highlighting its independent association with 30-day survival in ACS-related CS [7], even though further validation in a prospective clinical trial is warranted.

### Epidemiology

**Fig. 3:**
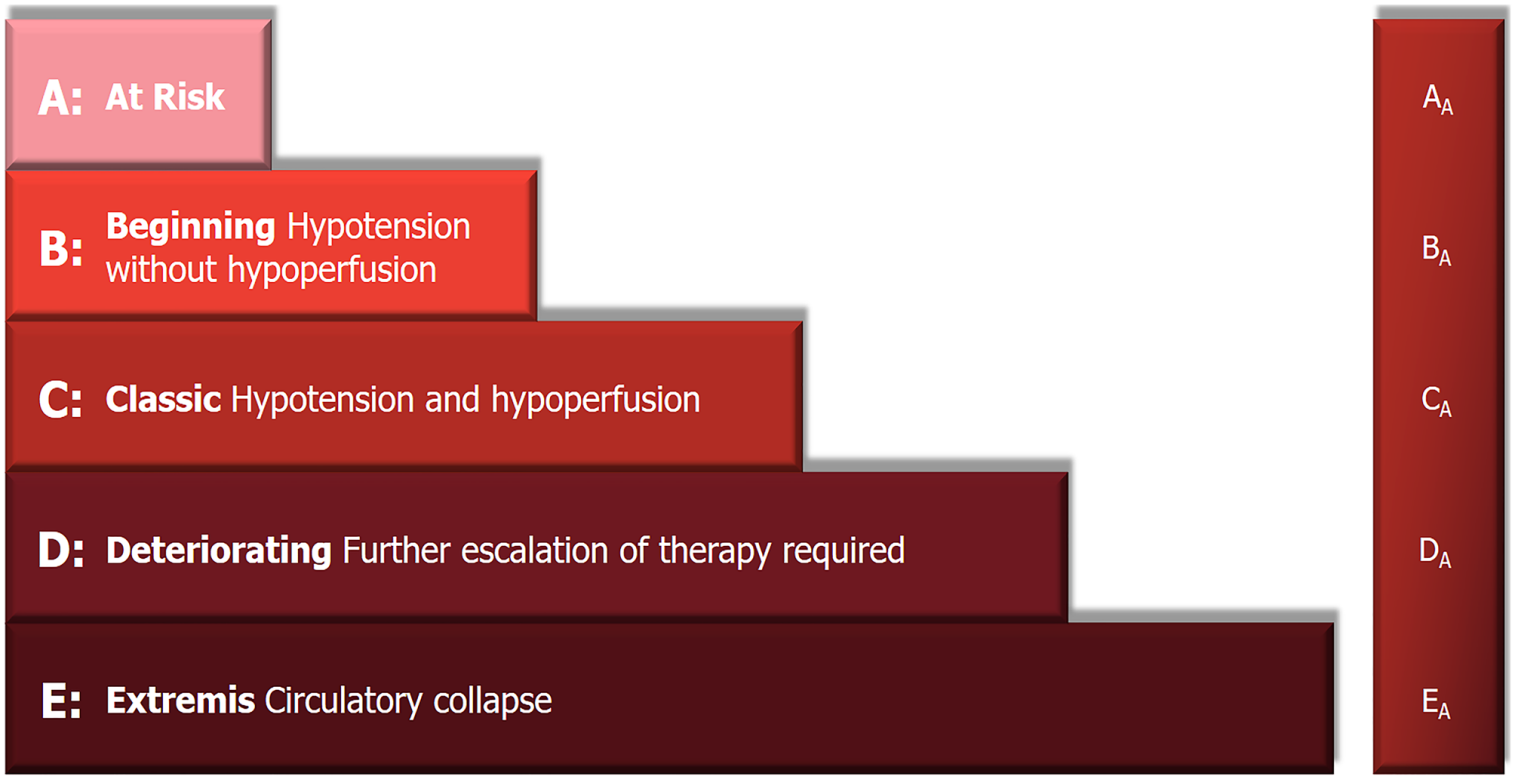
Readapted SCAI classification [6]

Figure 3 summarizes the most relevant epidemiological data on CS. ACS-related CS accounts for about 60–80% of the cases, while non-ACS-related CS for the remaining 20–40%, with small variation among studies [4, 9]. About the 70% of patients developing ACS-related CS presents with ST-elevation myocardial infarction (STEMI), while non-ACS-related CS encompasses a wide variety of diseases, ranging from acute decompensation of chronic heart failure, valvular heart disease, myocarditis and stress-induced cardiomyopathy [4, 6]. The incidence of ACS-related CS in Italy, according to the five main reported registries (BLITZ, IN-ACS Outcome, BLITZ-4, MANTRA e EYESHOT), is currently around 4.28% of total ACS [10].

The mortality of patients with ischemic CS is still high, ranging from 27% up to 51% [2], and their prognosis is critically dependent upon the time between the hospital admission and the start of revascularization procedure [11]. In fact, it has been estimated that 3.3 additional deaths per 100 percutaneous coronary intervention (PCI)-treated patients occur for every 10-min treatment delay [12]. Furthermore, survival of ACS-related CS appears to be influenced by the cardiac wall involved in the infarction [13]. Indeed, a recent study reported that in-hospital mortality of patients developing CS after inferior wall infarction was inferior compared to CS complicating anterior wall myocardial infarction, likely because of a lower left ventricular (LV) dysfunction due to a relatively smaller size of the involved cardiac area [14]. Despite the fact that inferior wall myocardial infarction could arguably involve the right ventricle and cause right ventricular failure leading to CS, this pathological event is the predominant cause of ACS-related CS only in 2.8% of cases, as attested by SHOCK (SHould we emergently revascularize Occluded Coronaries for cardiogenic shock) trial registry [15].

It has to be stressed that the development of CS in STEMI patients might occur either at hospital admission or within the next few days, named as early and late CS respectively, which are characterized by a similar mortality rate [16]. Identifying those patients who could develop late CS is particularly challenging since haemodynamic parameters at admission and lactate levels might not differ from those who will not develop this complication during hospital stay [16, 17]. Indeed, the pathophysiology is complex; the decline in LV ejection fraction (LVEF) and the reduction in blood pressure lead to a peripheral vasoconstriction which could eventually cause vasodilatation and inappropriate production of nitric oxide due to the triggered systemic inflammation [18]. The biomolecular pathways involved in systemic inflammation, neurohormonal activation and cardiac remodelling are known to be altered in CS especially when it recognises an ischemic aetiology. Having knowledge of this pathways is extremely helpful since it might provide insight into early markers of CS, optimizing risk stratification as well as therapeutic strategies.

### Therapeutic strategies

The management of CS starts with the investigation and recognition of the underlying cause. In fact, in ACS-related CS the cornerstone of treatment is urgent PCI, which, as mentioned above, represents the strongest prognosticator [11]. In both ACS-related and non-related CS, it is essential to provide respiratory support with non-invasive and invasive mechanical ventilation in case of acute hypoxemia or severe respiratory distress and to provide haemodynamic support, which can be achieved through pharmacological and non-pharmacological strategies. The first step is to assess and eventually withdraw drugs that may contribute to hypotension and the ones that exert a negative inotropic effect. The second step is to address reduced myocardial function as well as hypotension, through the use of inotropic and/or vasopressor agents (class of recommendation IIb according to the latest ESC guidelines) [19]. Despite the positive effects on haemodynamic profile and symptoms relief, the use of inotropes, such as dobutamine, epinephrine, levosimendan and milrinone, has been associated with increased mortality in several studies [20–22]. Several explanations to this finding have been proposed, such as increased incidence of arrhythmias, increased myocardial oxygen demand and possibly the fact that the need for inotropes might identify a subgroup of CS patients with more advanced heart failure [23]. Furthermore, to date, no study has shown a mortality benefit with the use of inotropes and/or vasopressor. Therefore, current evidence is inconclusive with regards to recommendation of one particular agents among inotropes and vasopressors [24]. In case of CS refractory to medical treatment, therapy escalation with non-pharmacological strategies, such as MCS, is advocated. In fact, the early use of MCS could convey a positive effect on these patients’ prognosis [25]. Therefore, it is particularly important to identify as soon as possible the patients that would benefit the most from an early invasive approach. However, these devices are not free from complications, especially infections, haemorrhagic and ischemic events. The most commonly used MCS devices include intra-aortic balloon pump (IABP), IMPELLA and veno-arterial extracorporeal membrane oxygenation (VA-ECMO). These devices have been investigated in several studies, even though often are relatively small and non-randomized trials, and a clear benefit in terms of both short- and long-term mortality has not been demonstrated. Table 2 shows the main trials that investigated the role of these devices in CS. For instance, the IABP-SHOCK II trial did not show any improvements in mortality rate at 30 days, 6 months and 1-year from admission with the use of IABP compared to medical therapy [3]. Furthermore, the IMPRESS in severe shock trial did not show any differences in mortality rate in CS patients assisted with IABP versus IMPELLA CP [26]. VA-ECMO used in CS has variable haemodynamic effects, in particular with regards to LV preload and afterload, which might make the patient’s response quite unpredictable. In fact, depending on both LVEF and peripheral resistance, the use of VA-ECMO could be associated with an increased LV afterload with LV distention that could in turn feed the vicious cycle and precipitate the clinical scenario. In this case, several strategies have been adopted to directly remove blood from the LV (venting) or indirectly reduce LV afterload. The most commonly used associations are VA-ECMO and IMPELLA and VA-ECMO and IABP. In fact, combining VA-ECMO with IABP has proven to be effective in reducing LV pressure and pulmonary oedema as well as in reducing mortality rates compared to VA-ECMO alone [27]. In addition to that, several studies have attested that VA-ECMO together with IMPELLA (ECPELLA) is associated with a lower 30-day mortality rate [28, 29], even though complication rates tend to be higher [29]. However, despite these data, evidence is still lacking on the best timing of MCS placement as well as how to accurately choose patients that could actually benefit from MCS offsetting the relatively high risk of complications.

**Table 2:** Main trials that investigated the role of mechanical circulatory support in cardiogenic shock

Study	Study population	Study information	Primary end-point	Results
ISAR-Shock (2008) [79]	26 patients with AMI-CS	Impella 2.5 vs IABP	Change in Cardiac Index from baseline to 30 min	Impella 2.5 improved haemodynamics Secondary end point 30-day mortality: no difference (46% both groups)
IABP-SHOCK II (2012) [3]	600 patients with AMI-CS and revascularisation	IABP vs MT	30-day mortality	No difference in 30-day mortality (39.7% IABP vs 41.3% MT)
Protect II Trial (2012) [80]	448 patients undergoing high-risk percutaneous intervention	IABP vs Impella 2.5	30-day mortality	No MAEs difference at 30 day Impella associated with decreased MAEs at 90 day
IMPRESS in severe Shock (2016) [26]	48 patients with STEMI-CS	Impella CP vs IABP	30-day mortality	No difference in 30-day mortality (50% Impella CP vs 46% IABP)
Pappalardo et al. (2017) [28]	157 patients with CS	VA-ECMO vs ECPella	In-hospital mortality	Lower in-hospital mortality with ECPella (47% vs 80%)
Russo et al. (2019) [27]	3997 patients with CS (meta-analysis)	VA-ECMO vs VA-ECMO + LV unloading (91.7% IABP)	All-cause mortality	Significantly lower mortality VA-ECMO with LV unloading (54% vs 65%)
Schrage et al. (2019) [81]	237 patients with IMPELLA for AMI-CS paired with 237 patients from IABP-SHOCK II trial	IMPELLA vs IABP	30-day mortality	No significant difference in 30-day all-cause mortality (48.5% versus 46.4%)
Patel et al. (2019) [82]	66 patients with CS	VA-ECMO vs ECPella	30-day mortality	Significantly lower mortality rate with ECPella (57% vs 78%)
Schrage et al. (2020) [29]	686 patients with CS	VA-ECMO vs ECMELLA	30-day mortality	Significantly lower 30-day mortality risk with ECMELLA (58.3% vs 65.7%)

*AMI-CS* acute myocardial infarction-related cardiogenic shock, *CS* cardiogenic shock, *ECMELLA* Impella support plus VA-ECMO, *ECPELLA* Impella support plus VA-ECMO, *IABP* intra-aortic balloon pump, *LV* left ventricular, *MAE* major adverse events, *MT* medical therapy, *STEMI-CS* ST-elevation myocardial infarction-related cardiogenic shock, *VA-ECMO* veno-arterial extracorporeal membrane oxygenation

### Prognostic markers

#### Biochemical markers

Well-established humoral markers of adverse outcome in CS include increased transaminase and creatinine levels [30], reflecting hepatic and renal hypoperfusion, and raised plasmatic arterial lactate which is considered an early marker of mitochondrial dysfunction and cellular impairment [31–33]. Another known marker is acidosis, which could lead to negative effects on myocardial contractility and impair the response to some vasopressors [34]. Recently, it has been demonstrated that a decrease in serum bicarbonate occurs earlier as compared to the raise of lactate levels, and low bicarbonate level might represent a stronger prognosticator of short-term mortality compared to high lactate level [35]. The development of acute kidney injury during CS is estimated at 13–28% [2] and is associated with longer in-hospital stay, cardiovascular events as well as mortality [36, 37].

In fact, several studies have found that acute kidney injury is an independent predictor of mortality in CS [38, 39]. Furthermore, evidence suggests that patients requiring renal replacement therapy have a higher mortality rate [38, 40]. The development of renal injury has been linked to biomarkers of nitric oxide/oxidative stress in STEMI patients and high-sensitivity C-reactive protein plasma levels [34]. The prognostic role of natriuretic plasmatic peptides has been widely recognised in both acute and chronic heart failure, in fact their levels correlate with LV dilatation, contractility and stiffness [41, 42]. In particular, natriuretic plasmatic peptide levels have been reported to be increased in STEMI patients developing CS as compared to non-complicated STEMI [43, 44]. In recent years, the prognostic role of soluble suppressor of tumorigenicity 2 (sST2) has emerged in the context of heart failure [45]. Tseng CCS et al. showed how sST2 levels increased in parallel with higher INTERMACS profile [46]. Regarding novel markers, pro-atrial natriuretic peptide, copeptid and mid-regional pro-adrenomedullin levels registered at admission in suspected STEMI patients showed to be independent predictors of late CS [47]. Other markers are currently under investigation, such as fibroblast growth factor-23 (FGF-23), high-sensitive C-reactive protein, angiopoietin-2 and soluble tumor necrosis factor receptor-1 (sTNFR1) [48, 49]. Moreover, the Optima CC trial has recently highlighted the role of circulating plasma dipeptidyl dipeptidase 3 [50], as it might predict either refractory CS and 90-day mortality [30, 50], even though these results come from a limited study population. Recent research is focusing on the role of micro-RNAs, due to accumulating evidence of their complex involvement in pathophysiological changes in heart disease [51], especially in advance heart failure, even though their prognosticator role in CS is still not completely clear. The identification of accurate and reliable biomarkers could be useful in the management of CS patients, since a quick prognostic stratification could be a valuable tool to guide the clinician to escalation therapy as well as markers to therapy response. In fact, future studies should focus on the change of both well-known and novel prognostic biomarker levels from hospital admission to 48/72 h since admission, since this could reflect the patient’s response to the adopted therapy, providing a positive feedback.

#### Echocardiographic parameters

**Fig. 4:**
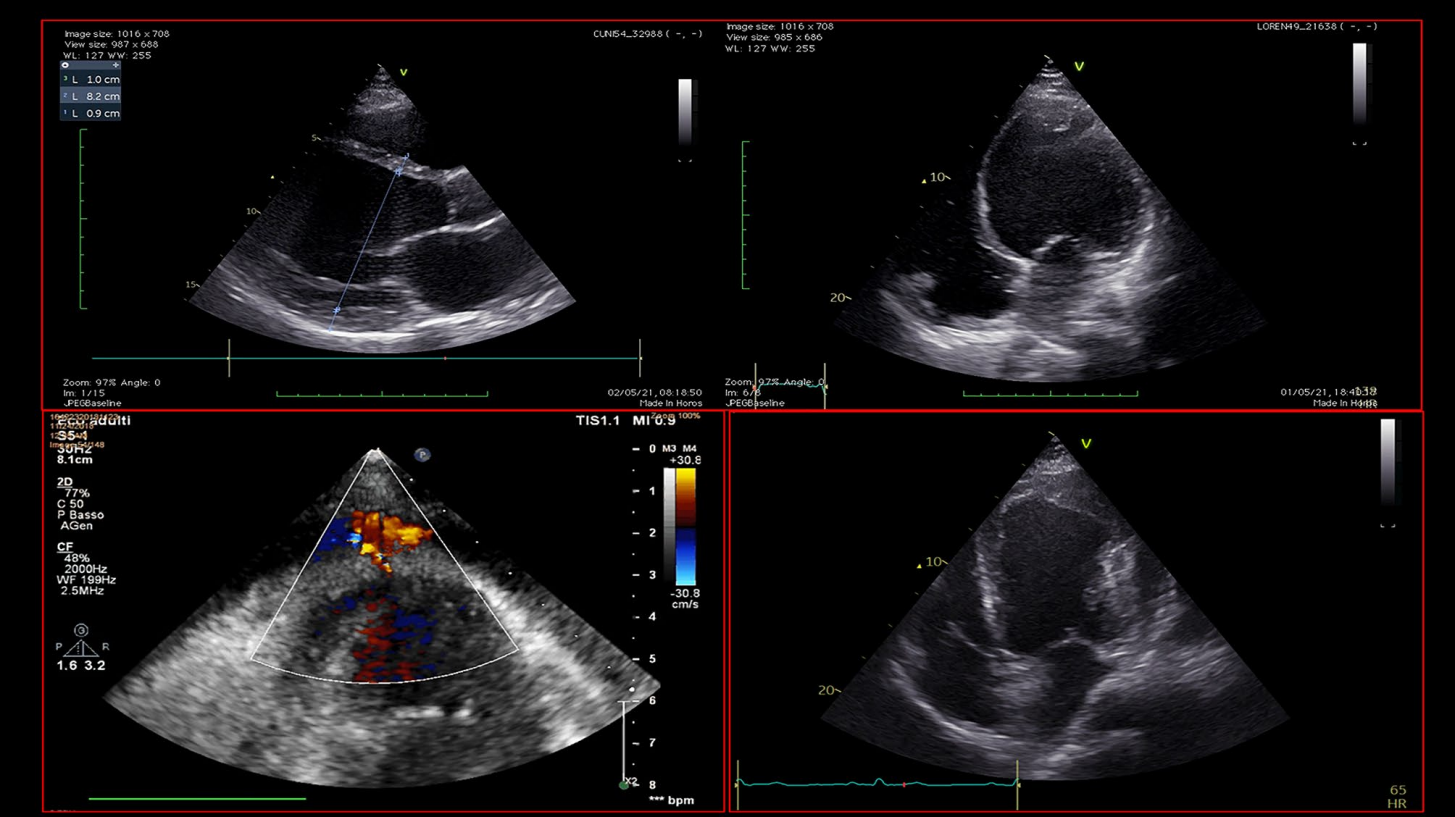
Echocardiographic assessment of patients admitted for cardiogenic shock. This picture shows different echocardiographic scenarios that can be found in patients admitted to intensive cardiac care unit for cardiogenic shock. The two images at the top show a severely dilated and impaired left ventricle with decreased wall thickness compatible with dilated cardiomyopathy, in presence of left ventricular thrombosis. The picture at the bottom left shows a left ventricular pseudoaneurism in a patient with a recent ST-elevation myocardial infarction, with flow passage demonstrated by Color Doppler. The picture at the bottom right shows a finding suspicious for left ventricular aneurism or pseudoaneurism, in presence of extensive thrombosis

**Fig. 5:**
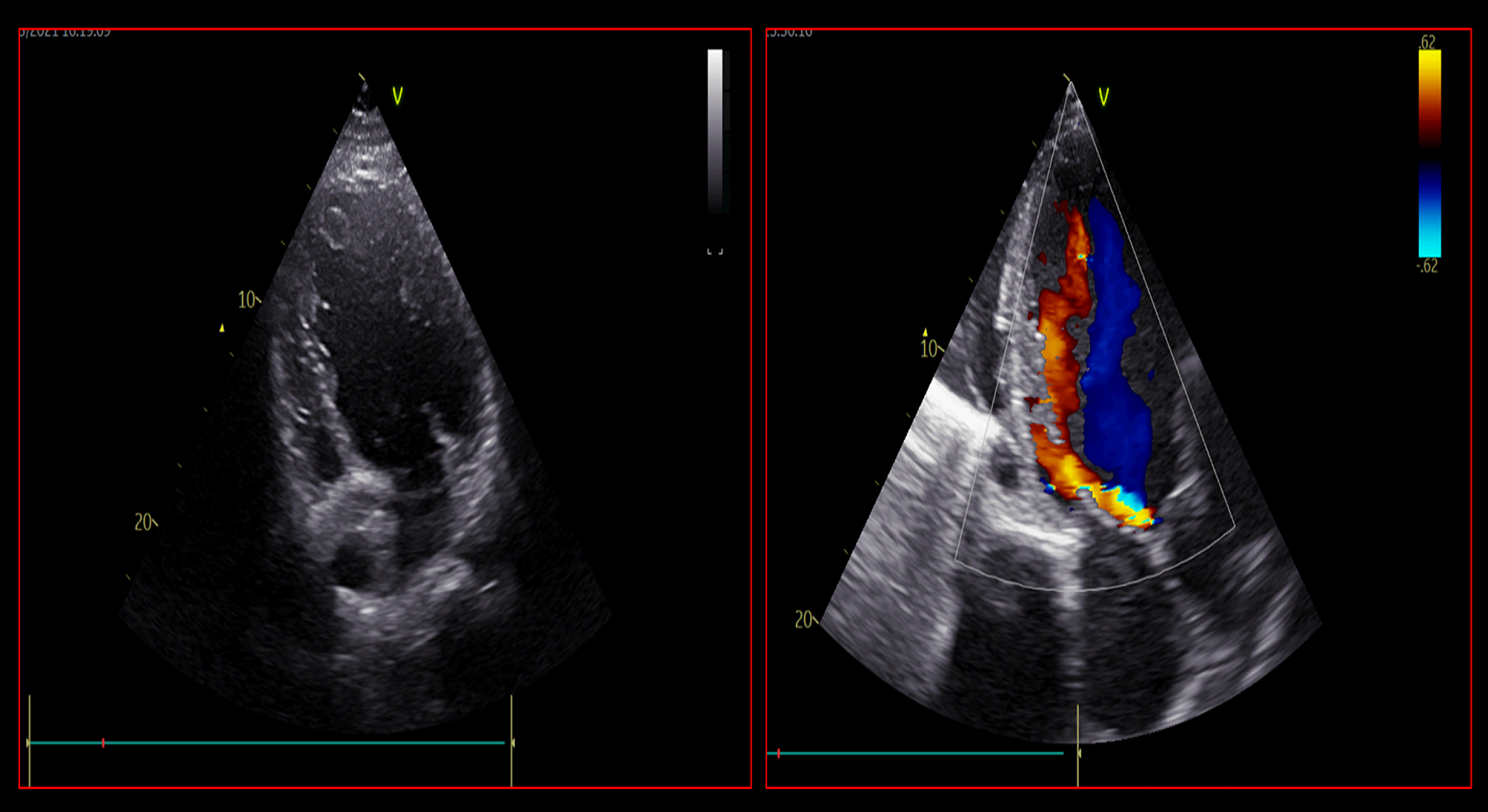
Echocardiographic assessment of possible contraindications to mechanical circulatory support. The picture on the left shows the presence of intracardiac thrombosis, localized at the apex of left ventricle. On the other hand, the picture on the right shows a case of significant aortic regurgitation with an eccentric jet, visualized in apical 3-chamber view. Both of these patients presented two possible contraindications to mechanical circulatory support placement

**Fig. 6:**
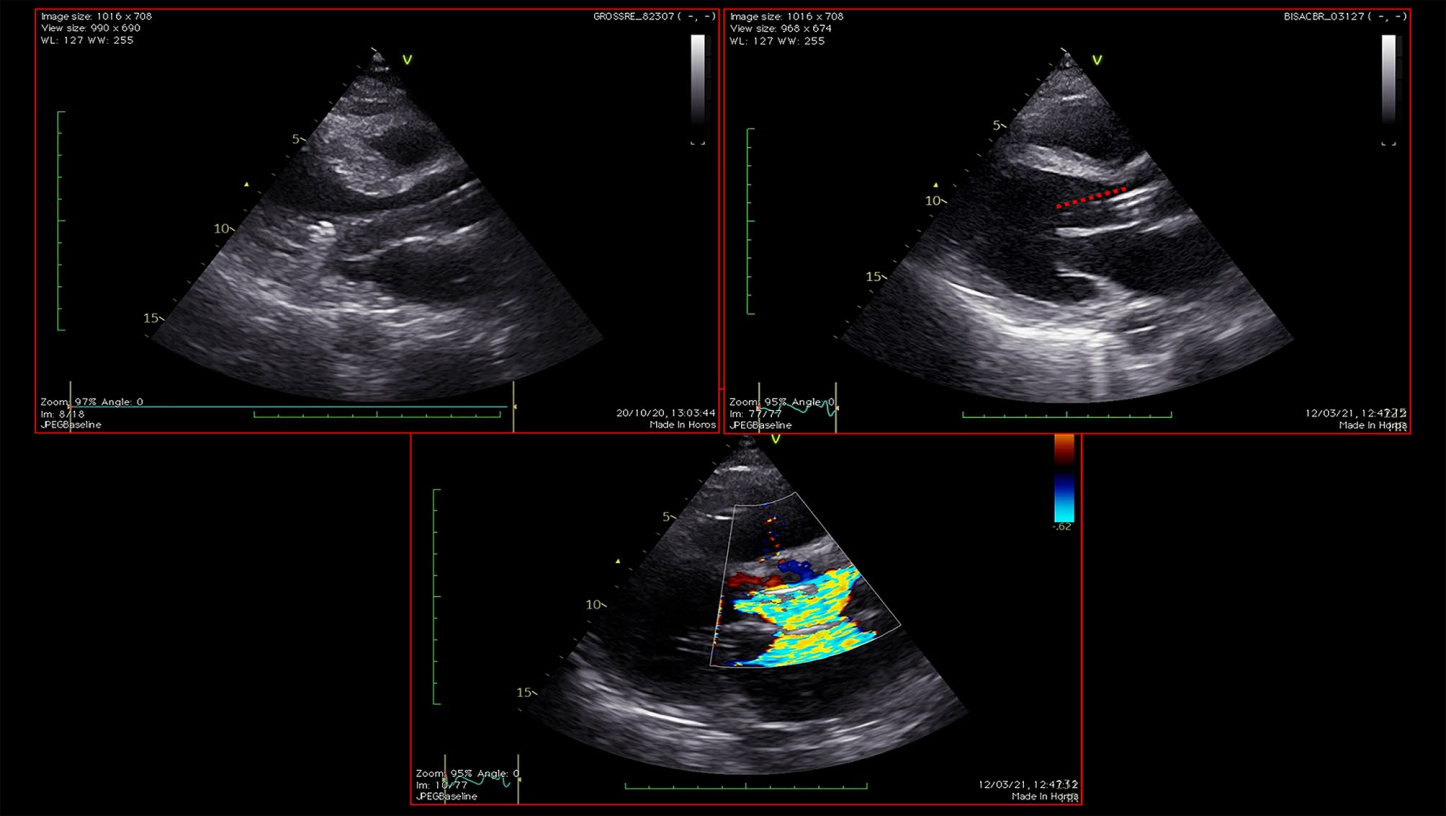
Echocardiographic assessment during mechanical circulatory support with Impella. This picture shows the echocardiographic assessment of cannula position during Impella support in two different patients. The distance from the inlet of the cannula and the aortic root, which should be around 35 mm in parasternal long axis view (top pictures), should be routinely measured in order to monitor cannula position and assess possible cannula dislocation. The picture at the bottom shows the outlet of the motor in the ascending aorta, visualized with the aid of Color Doppler

Echocardiography is a quick, highly available and first-line tool for a comprehensive evaluation of critically ill patients. Firstly, in non-ACS-related CS, it could help the clinician in the differential diagnosis of the variety of cardiac disorders that could cause CS (Fig. 4). In addition to that, it also provides a rapid haemodynamic and fluid status assessment, representing a valuable tool in patient’s monitoring. Its highest relevance is reached when MCS are contemplated as a therapeutic strategy. In fact, pre-MCS echocardiographic assessment is essential to identify possible contraindications to MCS, helping the clinician to choose the most appropriate device. In particular, the exclusion of intracardiac thrombosis as well as significant aortic regurgitation before placing MCS, especially VA-ECMO and IMPELLA, is of paramount importance (Fig. 5). Echocardiography, transesophageal echocardiography if transthoracic acoustic window is inadequate, has a crucial role both in ECMO cannulation as well as IMPELLA placement, since it guides the correct position of the cannulas (Fig. 6). A basal echocardiographic evaluation once MCS has been placed should always been obtained, in order to quickly detect any change in cannula position, worsening of aortic regurgitation, lack of aortic valve opening, increase in spontaneous echocontrast or endoventricular thrombosis. In VA-ECMO support, as mentioned above, echocardiography is useful to identify LV distention which is linked to increased LV afterload, prompting the adoption of LV venting strategies [28, 29]. Finally, it guides the weaning process from MCS, especially VA-ECMO, through the evaluation of LVEF amelioration, an increase in systolic S wave velocity at the lateral annulus of the mitral valve as well as the presence of LV outflow tract velocity time integral greater than 10 cm, which are all good predictors of successful weaning [52, 53]. Regarding the prognostic role of echocardiography in CS, data are scarcer. In fact, evidence is based mainly on standard echocardiographic parameters, first of all, LVEF. As mentioned above, LVEF has been included in the IABP-SHOCK II risk score as a predictor of outcome in ACS-CS patients [54]. However, only few studies have investigated the role of new echocardiographic techniques, such as speckle tracking echocardiography and 3-dimensional (3D)-echocardiography, which are less dependent from angle of insonation [55]. In fact, LVEF assessment strongly relies upon geometrical assumptions, and it is greatly influenced by load conditions, which might make it a less reliable parameter in the acute setting. It would be interesting to evaluate the prognostic role of LV global longitudinal strain (LV-GLS), as well as the role of left atrial strain and right ventricular free-wall longitudinal strain (RVFWSL) in CS patients. Both RVFWSL and LA volume have found to be independent predictors of cardiovascular events in heart failure with reduced LVEF, and in particular, RVFWSL resulted to be an independent predictor even in acute heart failure and in heart failure with preserved LVEF, as opposed to either RV-global longitudinal strain and LV-GLS [56]. Other studies have confirmed the prognostic role of RV dysfunction in HF [57], independently of LV function [58], as well as in various cardiovascular diseases [59]. On this matter, a recent investigation showed that 3D-echocardiography-derived RV-ejection fraction was associated with prognosis in patients with refractory CS treated with ECMO [60]. Since both myocardial strain analysis and 3D-echocardiographic assessment have been widely proven to successfully detect early myocardial alterations [55, 61, 62], it would be useful to investigate their role both in defining the optimal timing to escalation therapy as well as optimal timing for MCS weaning.

### Multiparametric prognostic scores

More complete prognostic scores have been formulated to help the risk stratification of CS patients in order to best allocate available resources and to promptly identify those patients that would benefit from more invasive therapeutic strategies in terms of survival and the optimal timing to proceed with MCS (Table 3). For instance, ECMO is able to provide pulmonary and circulatory supports for patients with refractory CS. However, it is characterized by a relatively high rate of complications, ranging from infections [63] to major bleedings [64]. Hence, a thorough evaluation of patients’ response predictor has to be performed in order to improve survival of CS requiring ECMO. In this direction, the SAVE (survival after veno-arterial ECMO) score proved able to stratify in-hospital mortality in this cohort, elaborated from registry data of 3846 CS patients [65]. The score included seven items: CS aetiology, age, body weight, organ failure pre-ECMO, ventilation, diastolic blood pressure before ECMO, pulse pressure before ECMO and plasma bicarbonate level before ECMO [65]. According to these parameters, the score classifies CS patients into five classes of increasing severity with corresponding survival rates of 75, 58, 42, 30 and 18%, respectively [65]. This score highlights the prognostic weight of the underlying cause of CS: transient events such as myocarditis are associated with a better outcome [66] as compared to ACS patients, as in the latter category only early PCI is essential for increasing survival [67]. Another score, the ENCOURAGE (prediction of Cardiogenic shock Outcome foR AMI patients salvaged by VA-ECMO) risk score has been developed from a smaller study population represented by 160 ACS-related CS patients in order to predict mortality during intensive care unit stay [68].

**Table 3:** Main prognostic scores in cardiogenic shock

SAVE score *[65]	ENCOURAGE score *[68]	CARD-SHOCK risk score [69]	IABP-SHOCK II score [64]
• CS aetiology • Age • Body weight • Organ failure* • Ventilation • Diastolic blood pressure* • Pulse pressure* • Plasma bicarbonate level*	• Age > 60 • Female sex • Body mass index > 25 kg/m^2^ • GCS < 6, creatinine > 150 μmol/L • Lactate (< 2, 2–8, or > 8 mmol/L) • Prothrombin activity < 50%	• Age • Previous myocardial infarction or CABG • ACS aetiology • LVEF < 40% • Lactate • eGFR	• Age • Plasma glucose level • Creatinine • Previous stroke • TIMI flow grade < 3 post-PCI • Lactate level

*ACS* acute coronary syndrome, *CABG* coronary artery by-pass, *eGFR* estimated glomerular filtration rate, *GCS* Glasgow coma score, *LVEF* left ventricular ejection fraction, *PCI* percutaneous-coronary intervention, *TIMI* thrombolysis in myocardial infarction

^*^Assessed before ECMO placement

Recently, other two risk scores have been published to predict short-term mortality of CS: CardShock risk score [69] and IABP-SHOCK II (Intra-Aortic Balloon Counterpulsation in Acute Myocardial Infarction Complicated by Cardiogenic Shock) risk score [54]. The CardShock risk score has been derived from a cohort of patients presenting with both ACS- and non-ACS-related CS [63] and includes seven items: age, previous myocardial infarction or coronary artery by-pass, ACS aetiology, LVEF < 40%, lactate and estimated glomerular filtration rate. Based on the score, patients can be grouped into 3 risk categories: low (scores 0–3), intermediate (scores 4–5) and high (scores 6–9) risk with a mortality rate of 8.7, 36 and 77%, respectively. According to this risk stratification, MCS such ECMO would be recommended in high-risk patients (mortality risk > 50%) [4]. On the other hand, IABP-SHOCK II risk score has been formulated based on a study population of patients with acute myocardial infarction undergoing PCI [54], and it considers six variables: age, plasma glucose level, creatinine, previous stroke, thrombolysis in myocardial infarction (TIMI) flow grade < 3 post-PCI and lactate level. IABP-SHOCK risk score classifies patients into three risk groups: low (scores 0–2), intermediate (scores 3–4) and high-risk (scores 5–9), with 30-day mortality rate of 23.8%, 49.2% and 76.6%, respectively [64]. In accordance to this score, CS patients with a high-risk class could be considered candidates for ECMO. Both these risk scores were characterized by a good predictive value when applied to a large cohort of real-world patients, including ACS and non-ACS subjects, even though their overall performance was higher in the ACS group [9].

As briefly presented in this paragraph, broad heterogeneity exists between these prognostic scores, making the decision of which one to choose quite challenging. First of all, some scores apply only to ACS-related CS whereas others have been validated in both CS etiologies, which make their application in clinical practice less easy. Furthermore, often enough different scores are based upon diverse clinical and laboratoristic parameters, which is another aspect to consider among the limitations of these scores. As mentioned above, the currently available prognostic scores lack innovative parameters, including novel biomarkers as well as novel echocardiographic indexes, derived from more advanced technologies. For instance, the echocardiographic assessment of myocardial strain could represent a more reliable parameter compared to LVEF, being able to identify earlier and smaller myocardial changes. This could be useful not only to promptly refer the patient to more advanced therapies but also to define the best timing to wean the patients from MCS, limiting the invasive support together with its risk of complications only to the necessary time to myocardial recovery. Based on these premises, new clinical trials are warranted to investigate the role of new markers in the risk stratification of patients with CS.

### Troubleshooting

- Due to the lack of demonstrated benefit in terms of survival in heart failure, the routine use of pulmonary artery catheter has been fallen out of favour [70–72]. However, recent evidence suggests that the use of pulmonary artery catheter in the early stages of CS might help the identification of the phenotype of CS and therefore guiding the following therapeutic strategies [73, 74]. In fact, the use of a Swan Ganz catheter at the patient’s bedside could be useful to obtain several haemodynamic parameters, such as central venous oxygen saturation, pulmonary capillary wedge pressure as well as pulmonary artery pressure. To date, there is no randomized clinical trial that assessed the utility of this invasive haemodynamic monitoring in a cohort of CS patients. Prospective registries and trials would be exetremely useful in order to define the role of pulmonary artery catheter in CS algorithms.
- The known prognostic scores in cardiogenic shock have still modest prognostic accuracy [75]. Furthermore, the same scores lack the inclusion of data derived from invasive haemodynamic monitoring and echocardiographic parameters obtained from advanced techniques, that could help further risk stratification. Future research should focus on designing a comprehensive prognostic score, that includes these parameters, facilitating the identification of those patients that could benefit from a more aggressive approach, including MCS, and those patients that should be addressed towards palliative care, after an appropriate multidisciplinary approach.
- Regionalized networks dedicated to CS should be designed and implemented. In fact, dedicated networks for time-dependent conditions, such as ST-segment elevation myocardial infarction, stroke and trauma network, have dramatically improved the prognosis of these patients [76, 77]. The collaboration between peripheral centres and level 3 centres, according to ‘hub and spoke’ model, is essential especially with regards to the centres dedicated to heart transplant and LVAD.
- As a consequence of the development of CS networks, in level 3 centres should be advocated the presence of shock team, consisting of dedicated cardiac intensivists and intensivists, in order to coordinate the most appropriate and time-effective therapeutic strategy. Furthermore, perfusionists and physiotherapists should also be included in a multidisciplinary CS team are as well as other professional figures with specific expertise in order to help minimize short-term as well long-term consequences of CS.

## Conclusions

The early identification of patients in the phase of ‘pre-shock’ or before the development of a refractory CS, by the elaboration of dedicated scores, is of paramount importance for the optimization of resources allocation. This can guide the clinician to a closer monitoring of this population, as well as it can accelerate its transfer to tertiary centres, where invasive therapies—such as MCS—are available. On the other hand, knowing the pathways involved in the development of refractory CS might encourage the advancement of therapies aiming at stopping or reducing the neurohormonal activation. That being said, it would be extremely useful to design new trials to elaborate new prognostic score, including the ones derived from advance imaging techniques as well as from invasive monitoring.
